# Differences between germline and somatic mutation rates in humans and mice

**DOI:** 10.1038/ncomms15183

**Published:** 2017-05-09

**Authors:** Brandon Milholland, Xiao Dong, Lei Zhang, Xiaoxiao Hao, Yousin Suh, Jan Vijg

**Affiliations:** 1 Department of Genetics, Albert Einstein College of Medicine, 1301 Morris Park Avenue, Bronx, New York 10461, USA; 2 Department of Ophthalmology & Visual Sciences, Albert Einstein College of Medicine, 1301 Morris Park Avenue, Bronx, New York 10461, USA; 3 Department of Medicine, Albert Einstein College of Medicine, 1301 Morris Park Avenue, Bronx, New York 10461, USA

## Abstract

The germline mutation rate has been extensively studied and has been found to vary greatly between species, but much less is known about the somatic mutation rate in multicellular organisms, which remains very difficult to determine. Here, we present data on somatic mutation rates in mice and humans, obtained by sequencing single cells and clones derived from primary fibroblasts, which allows us to make the first direct comparison with germline mutation rates in these two species. The results indicate that the somatic mutation rate is almost two orders of magnitude higher than the germline mutation rate and that both mutation rates are significantly higher in mice than in humans. Our findings demonstrate both the privileged status of germline genome integrity and species-specific differences in genome maintenance.

## Abstract (web summary)

Germline mutation rates are known to vary between species but somatic mutation rates are less well understood. Here the authors compare mice and humans, observing that somatic mutation rates were nearly two orders of magnitude higher in both species, with both mutation rates significantly higher in mice.

As first noted by Sturtevant^1^,^2^ the genetic material is mutable at a rate subject to natural selection. However, multicellular organisms also have a somatic genome with a mutation rate that is not necessarily similar to the germline mutation rate. While there is evidence that, in mammals, the spontaneous mutation rate in the germline is lower than in somatic cells^3^, thus far a direct comparison has not been made, due to the lack of reliable methods to measure somatic mutation frequencies in DNA from tissues and cell populations^4^. While a germline mutation will be present in all somatic cells, a post-zygotic, somatic mutation can only be detected when the cell gives rise to a lineage comprising a large fraction of the cell population sampled. Indeed, with the rapid increase of next-generation sequencing, postzygotic mutations have been detected in this way^5^,^6^,^7^,^8^,^9^, but such cases are only the tip of the iceberg and do not give a direct estimate of the somatic mutation rate.

In the past, somatic mutations in single cells have been detected at reporter loci^10^,^11^, but estimates of spontaneous mutation rates based on such surrogate genes cannot be considered as representative for the genome overall. Alternatively, it is now possible to sequence the genomes of multiple single cells after treatment with a mutagenic agent; the average mutation frequency of which provides an estimate of the effects of that agent^12^. However, to determine the true, spontaneous somatic mutation frequency in this way requires a well-validated procedure to amplify the genomes of single cells. Here we present the first direct comparison of mutation rates in human and mouse single somatic cells, which are compared with human and mouse *de novo* germline mutation rates. We found that the somatic mutation rate is much higher than the germline mutation rate in both humans and mice. We also found a less dramatic, but still large, difference in both germline and somatic mutation rates between the two species, with mice having a higher rate of somatic and germline mutations per cell division. Finally, we found that germline and somatic mutations in each species had distinct spectra. Our results indicate that both species and tissue type can direct the amount and type of mutations and implicate somatic mutations as a possible cause of aging.

## Results

### Germline mutation rates

**Figure 1:**
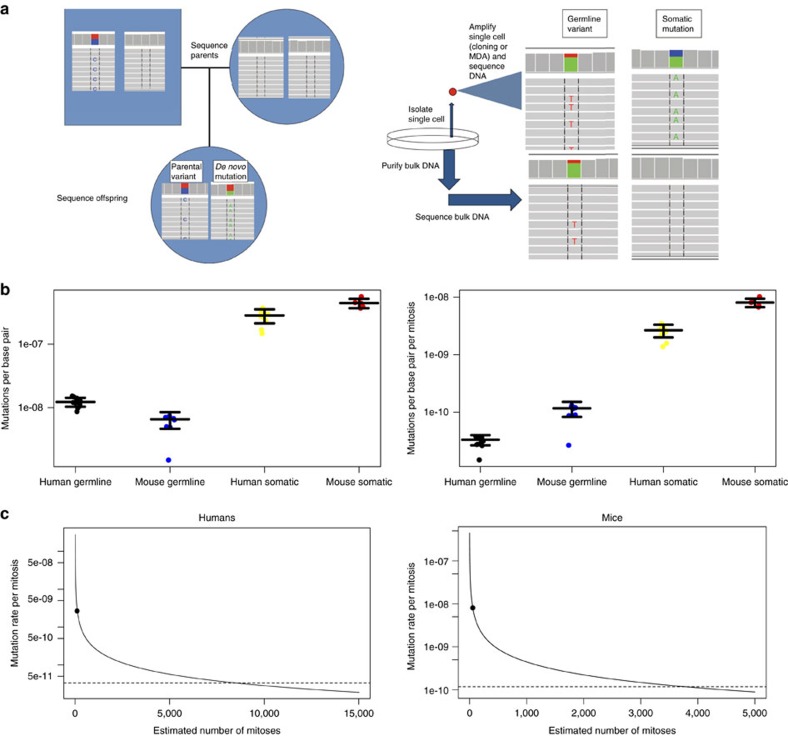
Direct comparison of somatic and germline mutation rates by high-throughput sequencing: experimental design and results. (**a**) Schematic representations of *de novo* germline (left) and somatic (right) mutation identification after whole genome sequencing. Germline mutations were determined by calling SNVs in DNA from offspring not present in parental DNA, while somatic mutations were identified as those present in single amplified fibroblasts or unamplified fibroblast clones, but not present in bulk DNA from the same cell populations. (**b**) Germline and somatic mutation frequencies in human and mouse before and after correction for the number of cell divisions. Horizontal bars indicate median ±1s.d. All groups were significantly different from all other groups (Wilcoxon test; mouse germline versus mouse somatic frequency: *P*=0.0016; mouse germline versus human germline frequency: *P*=1.6e-5; mouse germline versus human somatic frequency: *P*=4.6e-5; mouse somatic versus human germline frequency: *P*=0.00032; mouse somatic versus human somatic frequency: *P*=0.0013; human germline versus human somatic frequency: *P*=3.09e-6; mouse germline versus mouse somatic rate: *P*=0.0016; mouse germline versus human germline rate: *P*=0.0022; mouse germline versus human somatic rate: *P*=4.57e-5; mouse somatic versus human germline rate: *P*=0.00032; mouse somatic versus human somatic rate: *P*=0.00067; human germline versus human somatic rate: *P*=3.09e-6). (**c**) Number of somatic mitoses necessary to equalize the somatic and germline mutation rates in humans and mice, assuming the germline mutation rates are correct. The solid lines indicated the predicted somatic mutation rate for the given number of mitoses; the values used in the paper are indicated with large points. The dashed lines indicate the germline mutation rates. The human fibroblasts, given the somatic mutation frequency we observed, would have had to undergo more than 8,000 mitoses for the somatic mutation rate to be equal to the germline mutation rate. The mouse fibroblasts would have had to undergo over 3,000 mitoses to have the same mutation rate per mitosis as the germline cells.

Data on germline mutation frequency in humans was obtained from whole genome sequencing data of family trios obtained from ref. 13 and mutations reported in ref. 14; data on germline mutation frequency in mice was obtained using sequencing data from ref. 15 plus one C57BL/6 quartet, that is, parents and two offspring, which we sequenced ourselves (Fig. 1a; Methods; Supplementary Tables 1 and 2). In both the human and mouse datasets, *de novo* single nucleotide variants (SNVs) in offspring were called using three variant callers (Methods; Supplementary Fig. 1a). Germline mutations in the mouse quartet were verified using Sanger sequencing, which confirmed 75% of the mutations called (Supplementary Table 3). In humans, the frequency of germline mutations observed in the different trios was, on average, 1.2 × 10^−8^ mutations per base pair (bp), very similar to that reported previously^16^,^17^. For mice we found 7.0 × 10^−9^ and 6.7 × 10^−9^ mutations per bp for the two mouse pedigrees of our own and a mean of 5.3 × 10^−9^ mutations per bp (Fig. 1b) for the mouse pedigree data taken from ref. 15. Overall, we found a mean germline mutation frequency in mice of 5.7 × 10^−9^ mutations per bp, a number in reasonable agreement with the results of a long-term breeding study, which arrived at an estimate of 4.6−6.5 × 10^−9^ mutations per bp per generation^18^.

As most SNVs are a consequence of replication errors^19^, the raw *de novo* mutation frequencies were corrected for the number of cell divisions per generation, which differs greatly between humans and mice. As the germline mutation rate is predominantly determined by the male^20^, we used the values reported for the male germline in humans and mice^21^,^22^. After adjusting for the number of mitoses (Methods; Supplementary Table 4), we calculated a median germline mutation rate of 3.3 × 10^−11^ and 1.2 × 10^−10^ mutations per bp per mitosis for humans and mice, respectively. Hence, the mouse germline mutation rate per mitosis is over three-fold higher than that of humans (Fig. 1b).

### Somatic mutation rates

To determine somatic mutation frequencies in humans and mice we used early passage, primary dermal fibroblasts isolated from a 6-year old male human and cells of the same type from a 5-day old male C57BL/6 mouse (Fig. 1a). As mentioned above, somatic mutation frequency cannot be determined by sequencing total genomic DNA due to the very low-abundance of such mutations, which are unique to individual cells. Therefore, we determined spontaneous mutation frequencies in human and mouse primary fibroblasts by whole genome sequencing of multiple single cells after whole genome amplification. However, SNV calling in whole genome-amplified single cells is susceptible to errors associated with the cell lysis and amplification process. As reported elsewhere, we developed and validated a re-engineered multiple displacement amplification-based procedure to reliably amplify whole genomic DNA from single cells (Methods and^23^). Using this procedure we sequenced five single mouse fibroblasts, and included sequencing data of six human fibroblasts generated using the same method at the same time^23^. In addition, we also included whole genome sequencing data of four unamplified human fibroblast clones derived from single cells in the same population from which cells were taken for whole genome amplification^23^. Somatic SNVs in each single cell or clone were called against the whole genome sequencing data of unamplified DNA from the aggregate cell populations, representing the germline sequence (Fig. 1a), using three variant callers (Methods; Supplementary Fig. 1b), with the overlapping variants (∼7%) taken as high-fidelity somatic variant calls. The results indicate a median somatic mutation frequency of 2.8 × 10^−7^ and 4.4 × 10^−7^ per bp for human and mouse, respectively, more than an order of magnitude higher than the germline mutation frequency in both species (Fig. 1b).

The absolute numbers of SNVs observed in our human fibroblasts (that is, about 850) are somewhat lower than recently reported by Lodato *et al*.^24^ (about 1,500) for whole genome-amplified human neurons. However, these latter results were not validated through a direct comparison with unamplified clones. In our present study we did perform such a validation and no significant differences were found between the single human cells (amplified) and the clones (non-amplified), indicating the validity of our single-cell assay (Supplementary Table 4). Indeed, the estimated FDR among somatic mutations, which we adjusted for, was 0.3, only slightly higher than the estimated FDR among germline mutations, 0.25. Interestingly, a recent study^25^ on unamplified neuronal clones obtained through nuclear transfer found only about 100 SNVs per cell. The increased number of SNVs observed by Lodato *et al*.^24^ were mostly GC to AT transitions and could be due to cell lysis at elevated temperature, something we prevented by using a low-temperature protocol^23^. More recently, whole genome sequencing experiments using organoid technology resulted in very similar numbers of somatic mutations, several hundred per cell in colon and small intestine tissues from juvenile donors, as observed in our present study^26^. In yet another study between 10 and 30 mutations per cell line were found in the exomes of induced pluripotent stem cells derived from the clonal expansion of reprogrammed peripheral blood mononuclear cells^27^; these results correspond to roughly 500–1,500 mutations per genome, as found by previous studies of induced pluripotent stem cells^28^,^29^, with elderly donors accounting for the higher end of that range. Therefore, although the different tissue types make direct comparisons impossible, our results are in the same range as those found by other groups studying somatic mutations in clones derived from the *in vivo* situation.

As we did for the germline mutation frequencies, we also corrected the somatic mutation frequencies for the number of cell divisions between zygote and the target cells. Here, we could not rely on consensus estimates from the literature, so we arrived at our own estimates by incorporating information about the number of cells in the body, the homoeostasis of dermal fibroblasts after birth, and our observations of the cells during their brief time in culture (Methods; Supplementary Table 4). After correction for the difference in the number of cell divisions we found a somatic mutation rate of 2.66 × 10^−9^ and 8.1 × 10^−9^ mutations per bp per mitosis in humans and mice, respectively, still more than an order of magnitude higher than the corrected germline mutation frequencies in their respective species (Wilcoxon test: *P*=0.0015 in mice, *P*=3.09 × 10^−6^ in humans). Interestingly, the corrected somatic and germline mutation rates were significantly higher in mice than in humans (Wilcoxon test: *P*=0.0022 in the germline, *P*=0.00067 in the soma) (Fig. 1b).

This first direct comparison of germline and somatic mutation rates in two species indicate a more than one order of magnitude difference, with somatic cells much less capable of retaining the integrity of their genome as compared to germ cells, that is, sperm. It occurred to us that this difference could be due to an erroneous estimate of the number of cell divisions undergone by our somatic cells since the zygote. We considered the excess number of cell divisions between zygote and the fibroblasts analysed that would be necessary to equalize the somatic and germline mutation frequencies. This number is over 8,000 for human dermal fibroblasts and over 3,000 for the mouse dermal fibroblasts, hence, impossibly high (Fig. 1c). Thus, our findings are highly robust to even very large errors in the estimated number of mitoses.

### Mutation distributions and spectra

**Figure 2:**
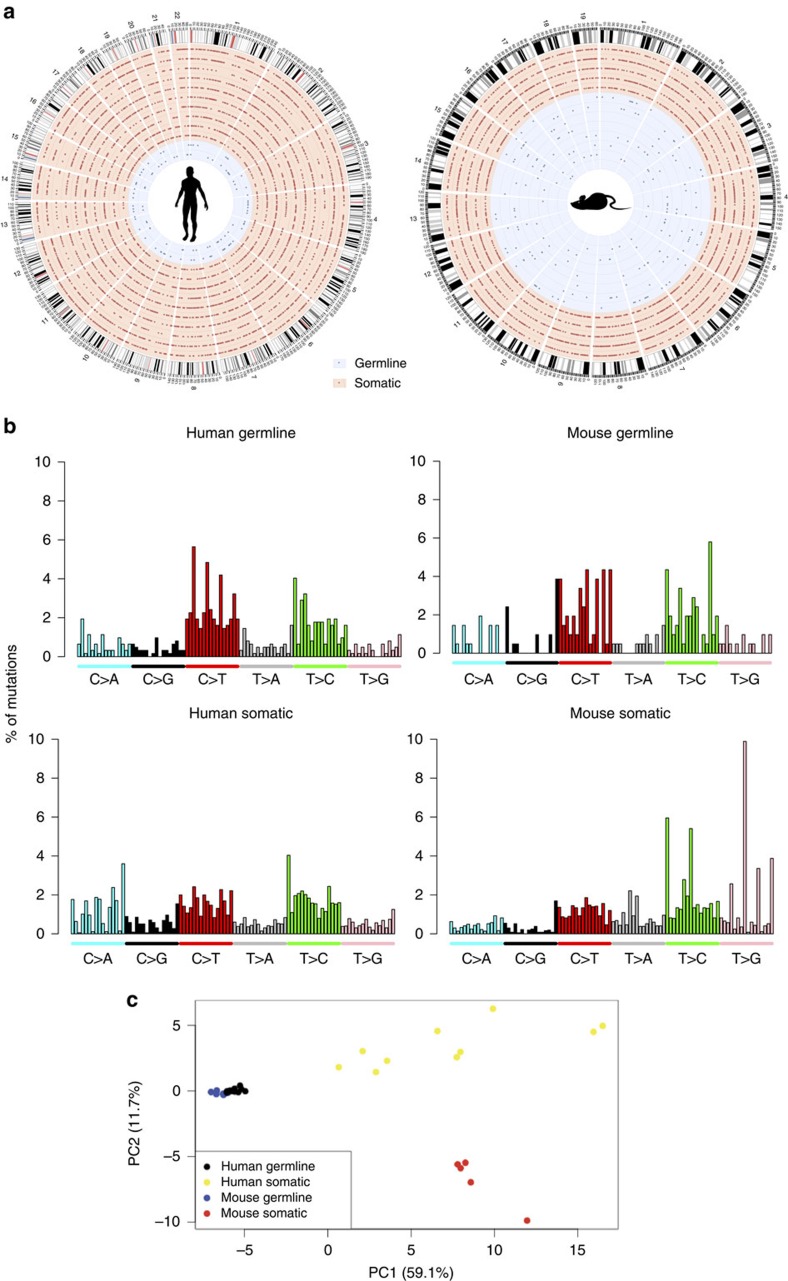
Distribution and spectra of human and mouse germline and somatic mutations. (**a**) Circos diagrams of mutations throughout the genome showing the genomic distributions of germline (blue) and somatic (red) mutations for which location data was available in humans (left) and mice (right). (**b**) Barplots of mutation types, including flanking bases, as a percentage of total mutations. (**c**) Principal component analysis of the data from **b** reveals distinct patterns of mutation that differ between germline and soma, as well as between mice and humans. Each point represents an individual offspring (in the case of germline mutations) or an individual cell (in the case of somatic mutations).

In both humans and mice, somatic and germline mutations were widely dispersed throughout the genome, appearing at many locations in every chromosome (Fig. 2a), but with distinct spectra of mutations (Fig. 2b). Principal component analysis of the spectra and trinucleotide context of mutations (Fig. 2c), showed that germline mutations in individual offspring tended to tightly cluster in a species-specific manner; by contrast, the somatic mutations in individual cells were more widely spread, suggesting a high degree of inter-cell heterogeneity in both humans and mice. However, somatic mutations in the two species were clearly separated from each other as well as from germline mutations, suggesting that the somatic mutation signature is species-specific. The first principal component, which appeared to separate germline and somatic mutations, was contributed to primarily (38.5%) by TA->CG and CG->TA mutations. Indeed, the proportion of CG->TA mutations was found to differ significantly between germline and somatic mutations after controlling for species (*P*=9.1 × 10^−7^, ANOVA, df=1, F=37.292, Table 1). The enrichment in CG->TA mutations among germline mutations is most likely a consequence of deamination of methylated cytosines. Sperm is one of the most highly methylated cell types, with over 80% of CpG sites being methylated^30^, and most germline mutations are thought to originate in the father^17^,^20^. The distinctive spectra of germline mutations in mice and humans may, therefore, reflect their unique epigenetic configuration.

The second principal component, which appeared to separate human and mouse somatic mutations, was mainly contributed to by CG->AT and TA->GC mutations; together, these mutations accounted for over 41% of its value. ANOVA confirmed that the proportion of TA->GC mutations was found to be significantly affected by species, whether the mutations were germline or somatic, and the interaction between those two factors (*P*=8.4 × 10^−7^, 3.9 × 10^−9^ and 7.3 × 10^−8^; df=1, 1 and 1; and F=37.60, 65.42 and 49.07 respectively; Table 1). The high enrichment of TA->GC mutations among mouse somatic mutations, a proportion nearly three-fold higher than in human somatic mutations, may be attributed to less effective repair of thymine dimers in mice; indeed, it has been known for decades that human cells are several times more effective in repairing photodimers than rodent cells^31^.

The distributions of germline and somatic mutations across different genomic features were similar (Table 2). In general, the mutations tended to reflect the overall composition of the genome, with the majority falling in either intergenic or intronic locations. If mutations were distributed randomly throughout the genome, then we would expect them to fall in exons 1.4% of the time in humans and 1.2% of the time in mice^32^. Compared to this expectation, there was no significant enrichment or depletion in the proportion of exonic mutations in mouse germline, mouse somatic, or human germline mutations. There did appear to be a significant depletion of exonic mutations among human somatic mutations (55/5,555, *P*=0.0085, two-tailed binomial test), but there were no significant differences in the ratios of nonsynonymous (Ns) to synonymous (S) mutations between any of the groups. The expected Ns/S ratio in the absence of selection depends on the codon usage in the species and the spectrum of mutations in the tissue, that is, 2.39 in the human germline, 2.76 in the human soma, 2.40 in the mouse germline, and 2.98 in the mouse soma. The Ns/S ratios observed were somewhat lower than these predictions (Table 2), indicating modest selection. This is in keeping with the fact that the mutational event and our observation of it are separated by only one generation (in the case of the germline mutations) or a few mitoses (in the case of the somatic mutations).

## Discussion

Our present results provide the first conclusive evidence that somatic mutation frequencies are significantly higher than germline mutation frequencies. Previously, this has only been suggested, based on data on somatic mutations using reporter genes^3^, but it has never been confirmed due to a lack of reliable assays for measuring low-abundance somatic mutations. The method we used here, single-cell whole genome sequencing after amplification, proved highly reliable, as indicated by the similar results obtained with unamplified DNA from clones.

The disparity in mutation rate between the germline and somatic tissues underscores the importance of genome maintenance in protecting the germline and dictating the disposable nature of the soma. Indeed, the latter has been considered as evidence that aging is caused by the accumulation of unrepaired somatic damage^33^. Different rates of somatic damage accumulation have been proposed to underlie species-specific differences in maximum life span^34^, which is in keeping with our present finding of a significantly higher mutation rate, both germline and somatic, in mouse as compared to human cells. The interspecies difference in mutation rate is consistent with our previous observations that both the level of expression and composition of DNA repair genes differ considerably between mice and humans^35^,^36^ and may point towards somatic mutations as a conserved mechanism of aging^37^. If, as has been suggested, each human baby has six new deleterious point mutations^4^, then each human somatic cell could have dozens, even hundreds, of deleterious mutations, and mice would have even more. Various ways by which species can cope with the occurrence of germline mutations have been proposed^38^, but much less research has addressed the manner by which organisms can cope with the much greater occurrence of somatic mutations. Further investigation of the biological mechanisms that permit proper cellular functioning in the presence of so many errors, and the way in which these mechanisms may eventually fail, should provide deeper insights into the biology of aging.

## Methods

### Sample preparation

Mouse dermal fibroblasts were obtained from a 5-day old male C57BL/6 mouse. All procedures involving animals were approved by the Institutional Animal Care and Use Committee (IACUC) of Albert Einstein College of Medicine. Human dermal fibroblasts from a 6-year old male human were provided by H. Choi (Seoul National University). The human fibroblasts were collected and protocols were approved as described in ref. 39^40^. Cells were grown in low glucose DMEM media containing 10% FBS, 100 IU ml-1 penicillin, 100 μg ml^−1^ streptomycin, 2 mM L-glutamine and 1% MEM non-essential amino acids (Gibco, Waltham, Massachusetts). Cultured cells were maintained at 37 °C with 10% CO_2_ and 3% O_2_.

### Germline and bulk DNA isolation and library preparation

DNA from cultured cell populations and mouse tail-clippings was isolated using the DNEasy kit (Qiagen, Venlo, Netherlands). DNA from the mouse quartet and bulk DNA from the cultured mouse fibroblasts was sequenced on the Illumina HiSeq 2500 after PCR-free library preparation at the Einstein Epigenomics Facility.

### Single cell collection and DNA amplification

Single cells were collected with the CellRaft system (Cell Microsystems, Research Triangle Park, North Carolina) and transported into 0.2-ml PCR tubes containing 2.5 μl PBS buffer. Single cell samples were frozen immediately on crushed dry ice and kept at −80 °C. For DNA amplification, 2.5 μl lysis buffer containing 400 mM KOH, 100 mM DTT, 10 mM EDTA, was added to a single cell in a PCR tube and kept on ice for 10 min. Then 2.5 μl stop buffer (400 mM HCl and 600 mM Tris–HCl) was added to the mixture. Finally, the master-mix containing PCR reaction buffer and Phi29 polymerase (REPLI-g UltraFast Mini Kit, Qiagen) was added. Amplification was carried out in a total volume of 41 μl for 1.5 h at 30 °C and then for 3 min at 65 °C.

### Single cell library preparation and sequencing

PCR-free libraries were prepared following the protocol for the Accel-NGS 2S DNA Library Kit (Swift Biosciences, Ann Arbor, Michigan). Briefly, using four incubations including two repair steps and two ligation steps, Illumina adaptor sequences were attached to the ends of fragmented double stranded DNA (dsDNA). Bead-based SPRI cleanups were used to remove oligonucleotides and small fragments. The resulting functional library was quantified by KAPA Library Quantification Kit (KAPA Biosystems, Wilmington, Massachusetts) and sequenced on the Illumina platform. The bulk samples were sequenced using Illumina HiSeq 2500 with 100 bp paired-end reads. The single cells amplified by ice lysis multiple displacement amplification were sequenced using Illumina HiSeq 2500 with 250 bp paired-end reads.

### Sequence alignment

Raw sequence reads were adaptor and quality trimmed using Trim Galore (http://www.bioinformatics.babraham.ac.uk/projects/trim_galore/) and aligned to reference genome human b37 and mouse grcm38 respectively using bwa mem^40^. PCR duplicates were removed using samtools^41^. The mapped reads were indel-realigned and base pair score quality recalibrated using GATK.

### Germline mutation calling

*De novo* germline SNVs from family trios were called using VarScan2 (ref. 42) and DenovoGear^43^ and Unifiedgenotyper^44^, using the default parameters and a minimum of × 20 coverage. Candidates were further filtered out if reported previously in dbSNP or if any variant-supporting read was present in either parent (Supplementary Fig. 1a). Germline SNVs were confirmed by Sanger sequencing (Supplementary Table 3).

### Somatic mutation calling

Somatic mutations were called using VarScan2 (ref. 42), MuTect^45^ and Unifiedgenotyper^44^ (Supplementary Fig. 1b). Briefly, somatic SNVs were called from a single cell or single cell clone using its corresponding bulk as control. For VarScan2 (ref. 42), we performed mpileup of bam files of single cell and bulk using samtools with default settings, and used ‘somatic' option of VarScan2 with a requirement of minimum sequencing depth of × 20. Mutations identified as ‘Somatic' by VarScan2 were taken for further filtration (Supplementary Table 5). For MuTect^45^, aligned bam file of single cell was input as ‘input_file:tumour' and bulk as ‘input_file:normal'. The dbSNP database and reference genome were included in its input parameters and all other parameters were set as default. SNVs identified as ‘Novel' and passed default filters by MuTect with minimum sequencing depth of × 20 in both single cell and bulk were taken for further filtration. For Unifiedgenotyper^44^, we called SNVs for each cell or bulk separately. The dbSNP database and reference genome were included in its input parameters and all other parameters were set as default. Low quality SNVs calls from Unifiedgenotyper were filtered out. Finally, we eliminated any mutations that were present in dbSNP or which had variant-supporting reads in the bulk. To avoid high false-positive mutation frequencies in the callsets of individual variant callers due to amplification errors and/or non-uniform coverage, the overlap among the three variant callers was taken as the final, high-quality mutation calls.

### Mutation rate estimation

Mutation rates were estimated by dividing the TPR and FDR adjusted mutation frequency by the estimated number of mitoses undergone by that cell type before sequencing (results summarized in Supplementary Table 4). We estimated the number of mitoses as the sum of the number of cell divisions during development, the number of cell divisions necessary to maintain homoeostasis of the tissue for the interval before the tissue was collected, and, since the somatic tissues sequenced were briefly grown in culture, the number of cell divisions in culture. Based on the most recent estimate of the number of cells in the human body^46^, 37 × 10^12^, we used log_2_(37 × 10^12^)=45.1 as the number of development mitoses; assuming that the weight ratio of 1:70,000 between humans and mice meant a similar ratio in the number of cells, we arrived at 29 mitoses for the mice. We used the reported turnover of skin cells^47^ to arrive at an estimate of 36.5 mitoses in humans; since the fibroblasts were taken from mice shortly after birth, we assumed they had under gone just one mitosis. Finally, based on our observations in culture, we estimated that the cells had undergone an additional 25 mitoses, giving final estimates of the number of somatic mitoses as 106.6 in humans and 55 in mice. Since the germline mutation rate has a strong male bias^17^,^20^, we considered only the number of mitoses in sperm cells. Based on the literature and the ages of our mice, we estimated a total of 56 germline mutations in the mice. For humans, we used the formula calculated for sperm cell divisions with age in humans and the exact ages of the fathers in our trios.

### Calculation of expected Ns/S ratio

To calculate the expected Ns/S ratio, we obtained the codon usage for each species^48^. Using this information, the probability that a given nucleotide substitution would or would not cause a change in protein sequence was calculated, and then multiplied by the prevalence of that mutation among somatic mutations in the relevant species and tissue. Finally, the calculated probability of a mutation being nonsynonymous was divided by the probability of a mutation being synonymous, giving the Ns/S ratio.

### Statistical analysis

Statistical analysis was performed using version 3.2 of R (ref. 49). Operating under the assumption that the minimum somatic mutation rate would be higher than the maximum germline mutation rate and the minimum mouse mutation rate would be higher than the maximum human mutation rate, sample size of human and mouse single cells was chosen to allow a statistically significant detection of differences between groups using the Wilcoxon test.

### Data availability

Raw sequence data was uploaded to the SRA under accession number SRP097734. A summary of datasets used can be found in Supplementary Table 1. All other data are available from the authors on reasonable request.

## Additional information

**How to cite this article:** Milholland, B. *et al*. Differences between germline and somatic mutation rates in humans and mice. *Nat. Commun.* **8,** 15183 doi: 10.1038/ncomms15183 (2017).

**Publisher's note:** Springer Nature remains neutral with regard to jurisdictional claims in published maps and institutional affiliations.

## Supplementary Material

Supplementary Information Supplementary Figure and Supplementary Tables

## Figures and Tables

**Table 1:** Mutation spectra.

	**% of mutations**				***P* value of effect on % of mutations**		
	**Human germline**	**Mouse germline**	**Human somatic**	**Mouse somatic**	**Human versus mouse**	**Germline versus somatic**	**Interaction**
CG->AT	9.78	7.89	19.68	7.10	0.0005	0.001	0.005
CG->GC	5.97	7.30	9.52	4.84	0.27	0.21	0.01
CG->TA	41.21	38.21	25.97	19.30	0.25	9.07E-07	0.54
TA->AT	9.68	8.79	8.03	13.02	0.89	0.29	0.005
TA->CG	27.50	31.43	28.40	30.62	0.06	0.78	0.42
TA->GC	5.87	6.38	8.41	25.12	8.4E-07	3.9E-9	7.3E-08

Germline and somatic mutation spectra in humans and mice.

*P* values calculated by ANOVA: df=1 for all comparisons; *F*=15.003, 12.665, 9.161, 1.248, 1.640, 7.336, 1.392, 37.292, 0.391, 0.016, 1.112, 9.406, 3.960, 0.077, 0.664, 37.60, 65.42 and 49.07.

**Table 2:** Genomic features.

	**Human germline**	**Mouse germline**	**Human somatic**	**Mouse somatic**
*% of mutations*				
3′ UTR	0.00	1.45	0.49	0.60
5′ UTR	0.00	0.00	0.07	0.11
downstream	0.89	0.00	0.65	0.90
exonic	2.68	0.97	0.96	1.23
exonic;splicing	0.00	0.00	0.00	0.03
intergenic	50.89	60.87	55.54	62.21
intronic	35.71	34.30	32.28	31.21
ncRNA	8.93	1.93	9.52	3.00
splicing	0.00	0.00	0.02	0.00
upstream	0.89	0.48	0.45	0.71
*% of exonic mutations*				
nonsynonymous	66.67	50.00	56.60	69.57
synonymous	33.33	50.00	37.74	28.26
stop gain	0.00	0.00	5.66	2.17
Observed Ns/S	2.0	1.0	1.65	2.54
Expected Ns/S	2.39	2.40	2.76	2.98

Distribution of mutations in genomic features and types of exonic mutations. As data on the locations of human germline mutations from ref. 14 were not available, only the mutations from ref. 13 were considered.
