# Supplementary material for: Differences between germline and somatic mutation rates in humans and mice
Source: Nat Commun. 2017 May 9;8:15183. doi: 10.1038/ncomms15183 (PMC5436103; doi:10.1038/ncomms15183)
Supplement: Supplementary Information — Supplementary Figure and Supplementary Tables [file ncomms15183-s1.pdf]

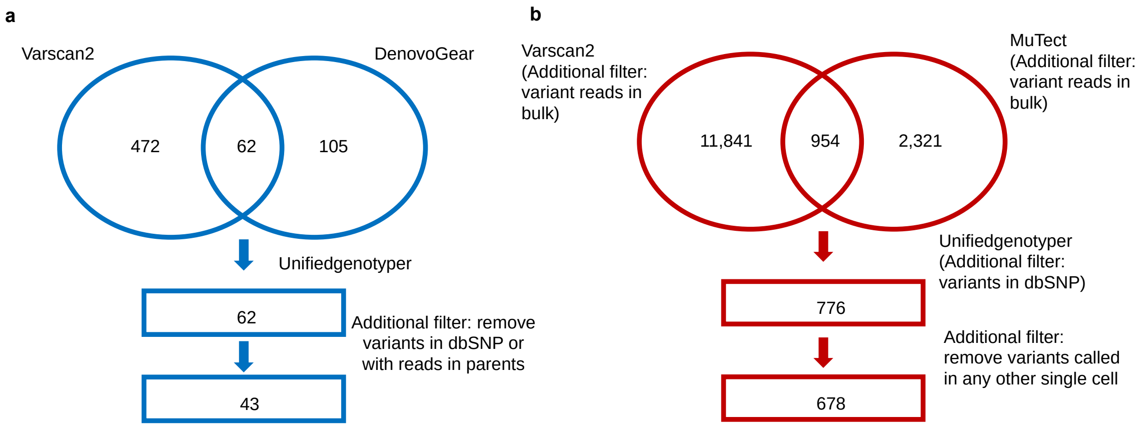

Supplementary Fig. 1. Detailed examples of mutation calling. (**a**) Germline mutation calling for sample F1. Only mutations found by both VarScan2 and DenovoGear were considered. Furthermore, variants not found by the GATK UnifiedGenotyper, present in dbSNP, or with variant-supporting reads in the parents were excluded. (**b**) Somatic mutation calling for sample MS.ice1. Only mutations bound by both VarScan2 and MuTect were considered. Furthermore, variants not found by the GATK UnifiedGenotyper, present in dbSNP or with variant-supporting reads in the bulk were excluded.

Supplementary Table 1. Summary of data sources

Sample ID	Species	Family ID	Sample Type	Source
BM1452.001	Human	hm01	Child	10.1002/humu.22814
BM1452.100	Human	hm01	Parent	10.1002/humu.22814
BM1452.200	Human	hm01	Parent	10.1002/humu.22814
BM1453.001	Human	hm02	Child	10.1002/humu.22814
BM1453.100	Human	hm02	Parent	10.1002/humu.22814
BM1453.200	Human	hm02	Parent	10.1002/humu.22814
HS.clone1	Human		Cloned dermal fibroblast	Dong, et. al., in press
HS.clone2	Human		Cloned dermal fibroblast	Dong, et. al., in press
HS.clone3	Human		Cloned dermal fibroblast	Dong, et. al., in press
HS.h24	Human		Dermal fibroblast single cell	Dong, et. al., in press
HS.h25	Human		Dermal fibroblast single cell	Dong, et. al., in press
HS.h2c	Human		Cloned dermal fibroblast	Dong, et. al., in press
HS.hdfcon1	Human		Dermal fibroblast single cell	Dong, et. al., in press
HS.hdfcon2	Human		Dermal fibroblast single cell	Dong, et. al., in press
HS.hdf2	Human		Dermal fibroblast single cell	Dong, et. al., in press
HS.hdf3	Human		Dermal fibroblast single cell	Dong, et. al., in press
MS.ice1	Mouse		Dermal fibroblast single cell	new dataset
MS.ice2	Mouse		Dermal fibroblast single cell	new dataset
MS.ice3	Mouse		Dermal fibroblast single cell	new dataset
MS.mdfcon1	Mouse		Dermal fibroblast single cell	new dataset
MS.mdfcon2	Mouse		Dermal fibroblast single cell	new dataset
L906F	Mouse	mm01	Parent	new dataset
L913M	Mouse	mm01	Parent	new dataset
M1	Mouse	mm01	Child	new dataset
F1	Mouse	mm01	Child	new dataset
NC_2	Mouse	mm02	Parent	10.1038/ncomms7684
NC_PF3	Mouse	mm02	Parent	10.1038/ncomms7684
NC_231	Mouse	mm02	Child	10.1038/ncomms7684
NC_232	Mouse	mm02	Child	10.1038/ncomms7684
NC_233	Mouse	mm02	Child	10.1038/ncomms7684
NC_3	Mouse	mm03	Parent	10.1038/ncomms7684
NC_PF5	Mouse	mm03	Parent	10.1038/ncomms7684
NC_351	Mouse	mm03	Child	10.1038/ncomms7684
NC_354	Mouse	mm03	Child	10.1038/ncomms7684
NC_358	Mouse	mm03	Child	10.1038/ncomms7684

Additional mutations from ref.[14], consisting of 10 sets of *de novo* mutations are excluded from this and the following tables as the mutations were taken from the paper and not generated bioinformatically.

Supplementary Table 2. Sequencing statistics

Sample ID	Average depth	Bases > 20x in both single cell and bulk / parents and offspring
HS.h24	26.5	1,648,051,466
HS.h25	26.9	1,465,262,548
HS.h2c	31.6	2,522,012,564
HS.HB	43.9	-
HS.hdfcon1	26.3	1,733,263,437
HS.hdfcon2	27.1	1,597,245,602
HS.hdf2	17.9	1,077,893,888
HS.hdf3	17.8	1,031,274,980
HS.clone1	29.5	2,513,956,859
HS.clone2	33.6	2,620,916,003
HS.clone3	27.6	2,452,906,052
MS.ice1	28.8	1,417,524,845
MS.ice2	29.4	1,353,229,979
MS.ice3	27.0	1,314,225,173
MS.mdfcon1	26.0	1,497,205,575
MS.mdfcon2	24.3	1,175,345,676
MS.mb	45.8	-
L913M	45.4	-
L906F	47.8	-
M1	45.2	2,324,972,805
F1	42.5	2,337,145,060
NC_2	23.1	-
NC_PF3	28.0	-
NC_231	23.7	1,635,675,992
NC_232	24.2	1,630,125,166
NC_233	23.9	1,621,709,947
NC_3	21.2	-
NC_PF5	20.5	-
NC_351	20.8	1,193,732,118
NC_354	22.0	1,227,069,432
NC_358	14.9	502,891,925
BM1452.001	28.5	2,081,722,080
BM1452.100	26.8	-
BM1452.200	28.1	-
BM1453.001	28.2	2,159,675,380
BM1453.100	27.2	-
BM1453.200	27.7	-

Supplementary Table 3. Results of Sanger sequencing

Sample	# callers reporting variant	Confirmed	Sequenced	% Confirmed
Human-clone1	3	3	3	100
Human-clone2	3	1	1	100
Human-h24	3	12	12	100
Human-h25	3	9	11	81.8
Human-hdf2	1	1	10	10
	2	4	13	30.8
	3	2	2	100
Human-hdf3	3	4	4	100
Mouse-ice1	1	0	14	0
	2	0	12	0
Mouse-trio-F1	1	0	12	0
	2	0	10	0
	3	8	10	80
Mouse-trio-M1	3	13	18	72.2

Supplementary Table 4. Estimates of mutation frequency and rate

Sample	Type*	Species†	Mutations called	Bases	TPR¶	FDR‡	Estimated mitoses	Mutation frequency	Mutation rate
ice1	S	M	678	1.42E+09	0.43	0.3	55.0	3.9E-07	7.1E-09
ice2	S	M	933	1.35E+09	0.43	0.3	55.0	5.6E-07	1.0E-08
ice3	S	M	729	1.31E+09	0.43	0.3	55.0	4.5E-07	8.2E-09
mdfcon1	S	M	681	1.50E+09	0.43	0.3	55.0	3.7E-07	6.7E-09
mdfcon2	S	M	641	1.18E+09	0.43	0.3	55.0	4.4E-07	8.1E-09
F1	G	M	43	2.30E+09	1	0.25	56.0	7.0E-09	1.3E-10
M1	G	M	41	2.29E+09	1	0.25	56.0	6.7E-09	1.2E-10
nc-231	G	M	28	1.64E+09	1	0.25	56.0	6.4E-09	1.1E-10
nc-232	G	M	32	1.63E+09	1	0.25	56.0	7.4E-09	1.3E-10
nc-233	G	M	29	1.62E+09	1	0.25	56.0	6.7E-09	1.2E-10
nc-351	G	M	16	1.19E+09	1	0.25	56.0	5.0E-09	9.0E-11
nc-354	G	M	16	1.23E+09	1	0.25	56.0	4.9E-09	8.7E-11
nc-358	G	M	2	5.03E+08	1	0.25	56.0	1.5E-09	2.7E-11
Clone1	S	H	906	2.51E+09	0.46	0.05	106.6	3.7E-07	3.5E-09
Clone2	S	H	876	2.62E+09	0.46	0.05	106.6	3.5E-07	3.2E-09
Clone3	S	H	349	2.45E+09	0.46	0.05	106.6	1.5E-07	1.4E-09
h24	S	H	574	1.65E+09	0.39	0.33	106.6	3.0E-07	2.8E-09
h25	S	H	562	1.47E+09	0.47	0.27	106.6	3.0E-07	2.8E-09
h2c	S	H	651	2.52E+09	0.46	0.05	106.6	2.7E-07	2.5E-09
hdfcon1	S	H	391	1.08E+09	0.43	0.3	106.6	3.0E-07	2.8E-09
hdfcon2	S	H	299	1.03E+09	0.43	0.3	106.6	2.4E-07	2.2E-09
hdf2	S	H	360	1.73E+09	0.43	0.3	106.6	1.7E-07	1.6E-09
hdf3	S	H	534	1.60E+09	0.43	0.3	106.6	2.7E-07	2.6E-09
BM1452.001	G	H	62	2.08E+09	1	0.25	425.1	1.1E-08	2.6E-11
BM1453.001	G	H	50	2.16E+09	1	0.25	586.1	8.7E-09	1.5E-11

* S=somatic; G=germline

† M=mouse; H=human

¶ TPR (sensitivity) for single cells, TPR is estimated using a kindred cell and clone combination (Dong et al., accepted); for germline, TPR is approximated as 1 because we found almost no Mendelian inheritance errors

‡ FDR (false discovery rate) is approximated using both Sanger sequencing and kindred samples

Supplementary Table 5. Germline SNPs called and missed by VarScan

Sample	Species	Total	Missed	Sensitivity	Type
mb-ice1	Mouse	60402	10228	83.07%	Single cell
mb-ice2	Mouse	53542	12423	76.80%	Single cell
mb-ice3	Mouse	53749	10147	81.12%	Single cell
mb-mdfcon1	Mouse	59044	9875	83.28%	Single cell
mb-mdfcon2	Mouse	48373	9059	81.27%	Single cell
hunamp-clone1	Human	2116163	30921	98.54%	Clone
hunamp-clone2	Human	2193044	32252	98.53%	Clone
hunamp-clone3	Human	2056154	29411	98.57%	Clone
hunamp-h2c	Human	1982400	39157	98.02%	Clone
hunamp-h24	Human	1208695	63030	94.79%	Single cell
hunamp-h25	Human	1089932	259466	76.19%	Single cell
hunamp-hdfcon1	Human	725396	56484	92.21%	Single cell
hunamp-hdfcon2	Human	662951	40499	93.89%	Single cell
hunamp-hdf2	Human	1305829	47187	96.39%	Single cell
hunamp-hdf3	Human	1166095	233600	79.97%	Single cell
